# Post-traumatic stress disorder (PTSD) among Filipino boys subjected to non-therapeutic ritual or medical surgical procedures: A retrospective cohort study

**DOI:** 10.1016/j.amsu.2019.04.004

**Published:** 2019-04-25

**Authors:** Gregory J. Boyle, Samuel Ramos

**Affiliations:** a University of Melbourne, Parkville, VIC, 3010, Australia; b Bond University, Gold Coast, QLD, 4229, Australia

**Keywords:** PTSD, Ritual genital cutting (Tuli), Medical circumcision, Trauma, Stress

## Abstract

In the Philippines, non-therapeutic genital cutting is viewed as a culturally sanctioned rite of passage from boyhood to manhood. Strong social and peer pressure is exerted on boys aged between 8-16years to submit to destructive genital cutting, despite the fact that many men who have been subjected to genital cutting during infancy or childhood often describe their experiences in the language of violence, torture, mutilation, and sexual assault. Among a group of 505 Filipino boys subjected to ritual genital cutting (Tuli), 69% fulfilled the DSM-IV criteria for a diagnosis of PTSD, while among 1072 boys circumcised by medical operators or their assistants, 51% exhibited PTSD symptoms. Pursuant to ritual genital cutting, almost 3 out of every 4 boys exhibited PTSD-like symptoms.

## Highlights

• 1072 Filipino boys (11–16 years) from the Batangas had undergone medical circumcision and 505 ritual circumcision (Tuli).
• 69% of the Tuli group exhibited PTSD symptoms and 51% of boys circumcised by medical operators exhibited PTSD symptoms.
• Boys subjected to Tuli exhibited PTSD comparable to Vietnam veteran inpatients at St. Cloud (Minnesota) VA Medical Center..
• Both groups reported higher PTSD (p < .0001) than women subjected to “very distressing or terrifying” obstetric procedures.
• A limitation of the study was its failure to examine ongoing levels of PTSD in both groups over increasing time intervals.

## 1 Introduction

In the Philippines, a largely Catholic country, traumatic genital cutting (circumcision) is regarded as a culturally sanctioned rite of passage from boyhood to manhood resulting in significant PTSD-like symptoms among much of the male population [[1], [2], [3], [4]]. PTSD can be loosely defined as the experience of major stress subsequent to a traumatic event sometimes leading to psychological fragility and instability. Strong social and peer pressure is exerted on boys aged between 8 and 16 years to submit to destructive genital cutting, despite the fact that men who have been subjected to genital cutting during infancy or childhood often describe their experiences in the language of violence, torture, mutilation, and sexual assault [[5], [6], [7]]. This PTSD retrospective cohort study is being revisited here in order to provide significant statistical results not included in the initial rather obscure report [2], relating to PTSD among boys subjected to genital cutting, which appear not yet to have entered the general medical and psychological literature.

Despite ethical concerns about the child's right to bodily integrity [8], and the fact that the Catholic Church itself has denounced non-therapeutic circumcision of normal, healthy boys [9], cultural pressure, harassment, bullying and intimidation evidently play a synergistic role in promoting the continuing non-therapeutic mass genital cutting of Filipino boys. Despite this cultural custom, the foreskin itself is comprised of highly specialized erogenous nerve endings [10,11], it protects the sensitivity of the glans penis, and it also plays an important functional role in facilitating sexual intercourse for both males and their female partners by reducing abrasive friction and resultant pain and discomfort [12,13].

Ritual genital cutting (Tuli or pukpok) typically occurs on *Sabado de Gloria* (Black Saturday) in a remote wooded area from which girls are excluded [14]. Typically, an elderly man (manong) stretches the boy's foreskin over a wooden anvil (tree stump), places a “cut-throat” barber's razor (labaha) lengthwise on top of the foreskin and then with a few quick blows slices the foreskin wide open, thereby exposing the glans. The resultant traditional dorsal slit (superincision), while damaging in and of itself, at least spares thousands of erogenous nerve endings and highly specialized sensory receptors located within the inner foreskin. The newly-cut boys then bathe in the cool water of a nearby river or stream in order to lessen the pain, and they also apply chewed guava leaves to the genital wound in an attempt to control bleeding and promote more rapid healing [[14], [15], [16], [17], [18]].

The Philippines Department of Health has launched a widespread public awareness program to discourage ritual cutting and subsequent bathing in a river, due to the high risk of contracting a tetanus infection with an open wound [19,20]. Consequently, many Filipino boys now undergo non-therapeutic genital cutting as part of mass ”ceremonies” at the hands of medical practitioners (in violation of ethical medical conduct and the Hippocratic Oath – *primum non nocere* − “*first do no harm*”) or even by their untrained volunteer assistants (often performed on unhygienic tables set up in school halls whereby quality control and sterile conditions cannot be assured. For example, with the express intention and clearly unethical aim of getting into the *Guinness Book of World Records*, 1500 boys were circumcised on the same day in Marikina City in 2011) [21]. Not counting the large number of likely botched circumcisions (including severe mutilations), non-therapeutic genital cutting under these crude circumstances may involve: (1) cutting a dorsal slit in the foreskin (akin to traditional “*Tuli*”), (2) making a V-cut (two intersecting diagonal cuts whereby a triangular section of the double-layered inner and outer foreskin is excised, thereby extirpating thousands of highly erogenous nerve endings and permanently reducing sexual sensation), or (3) actual circumcision itself whereby the foreskin is stretched forward over the glans and completely sliced off (“German cut” − whereby the exposed glans gives the appearance of a German military helmut). Understandably, many boys cry out in pain for analgesia and/or for their mothers to comfort them [2,3]. The traditional Tuli dorsal slit at least preserves most of the erogenous nerve endings and sensory receptors located on the inner surface of the foreskin [10,11]. In contrast, rather than being advantageous, most circumcisions performed by medical practitioners are even more destructive, excising most of the inner foreskin erogenous tissue, severely and permanently reducing sexual sensation, and thereby weakening sexual sensation and the ability to perform sexually, often leading to premature ejaculation (with little/no sexual sensation) and/or subsequent erectile dysfunction in later adulthood [12,[22], [23], [24], [25], [26], [27]].

In order to investigate PTSD-like symptoms among healthy Filipino boys undergoing non-therapeutic ritual genital cutting (*Tuli*) as compared with boys subjected to non-therapeutic medically-performed genital cutting procedures, a large empirical study was conducted in the Batangas Province of the Philippines [2,3]. PTSD levels (measured via the Watson et al. PTSD survey) [28] were found to be significantly elevated following both ritual Tuli and medically-performed circumcisions [2]. Given the direct association between PTSD and resultant suicidality [29], this large-scale PTSD study conducted in the Philippines is revisited here in view of the very high incidence of suicide among traumatized (circumcised) Filipino males where the “age-standardized suicide rate” (in 2010) per 100,000 head of population in the Philippines was reported as being 5.7 for males, but only 2.3 for females [30].

## 2 Method

### 2.1 Participants and procedures

Altogether, 3253 boys aged between 11 and 16 years from the general male population from five different schools were invited to participate in the genital cutting induced PTSD cohort study (Table 1). After obtaining all necessary permissions, biographical data was collected using a self-report survey questionnaire that included the boys' present age, their history of any prior traumatic experiences, age at time of genital cutting, the specific genital cutting method used (ritual vs. medical), motivation for submitting to non-therapeutic genital cutting, and the boys’ perceptions of their traumatic experience. The study was conducted and reported in line with STROCSS criteria [31].

**Table 1:** Demographic data for boys subjected to ritual “Tuli’ genital cutting vs. boys subjected to genital cutting by medically-trained personnel.

Biographical Information		Circumcision Procedure N = 1577	
		Medical n = 1072	Ritual n = 505
Participants Age (years)	<13	30.9%	17.8%
	14	22.1%	21.3%
	15	17.8%	24.3%
	16	27.1%	36.6%
Age of Circumcision (years)	<10	28.1%	18.6%
	11	28.6%	15.8%
	12	33.3%	35.4%
	>13	10.0%	30.2%
Feelings before the procedure	Fear	81.3%	89.9%
	Anger	17.3%	8.1%
	Others	1.4%	2.0%
Motivation	Social	60.0%	56.0%
	Religion	21.4%	34.1%
	Medical/Health	17.8%	8.7%
	Others	.8%	1.2%

Research registration: ACTRN12619000589189. http://www.anzctr.org.au/TrialSearch.

Altogether, 1577 boys met the criteria for inclusion in the study (i.e., aged 11–16 years when surveyed with no previous psychological trauma or pre-existing PTSD symptoms) [2,3]. Among those eligible, 1072 boys were subjected to medically performed genital cutting, while 505 boys were subjected to ritual Tuli. Among the boys subjected to circumcision by medical practitioners or their assistants, 60% did so due to social and peer pressure, 21.4% because of their religious beliefs, 17.8% for alleged but misguided medical/health reasons and 0.8% for other unstated reasons. Some 81.3% of boys reported experiencing fear and anxiety, 17.3% reported anger, while 1.4% reported other negative emotions. Among the boys subjected to ritual Tuli, 56% reported submitting to the destructive ritual cutting because of social and peer pressure, 34.1% because of their religious beliefs, 8.7% for supposed health reasons and 1.2% for other unstated reasons. Following ritual Tuli, 89.9% reported negative emotions of fear and anxiety, 8.1% reported anger, while 2% reported other negative feelings.

All participants completed the psychometrically valid and reliable Watson et al. PTSD-I interview rating scale [28]. The same questionnaire and PTSD interview survey was administered to both groups thereby enabling direct between-groups comparisons on the dependent variables. The Watson et al. PTSD-I scale comprises 17 items corresponding with the DSM-IV defined symptoms of PTSD [1], including Symptom A (threat to physical integrity of self); Symptom B (trauma-re-experiencing); Symptom C (avoidance of trauma inducing stimuli and numbing of responsiveness); and Symptom D: increased physiological arousal. Symptom E (duration more than 1 month) and Symptom F (impairment in social and/or occupational functioning). The boys responded to each item on a 7-point Likert-type scale ranging from 1 (“no or never”) to 7 (“extremely or always”).

## 3 Results

Among the ritual genital-cutting (Tuli) group, 69% fulfilled the DSM-IV criteria for a diagnosis of PTSD [1], while among the medically circumcised boys, 51% exhibited similar PTSD-like symptoms. Thus, nearly three out of every four boys exhibited discernible PTSD-like symptoms pursuant to their being subjected to ritual genital cutting. Although not included in the earlier symposium proceedings [3], the 505 boys subjected to ritual Tuli exhibited significantly higher PTSD levels than did the 1072 boys circumcised by medical operators [OR = 2.13, 95% CI = 1.70–2.66, χ2(df = 1) = 43.74, p < .0001] [2].

## 4 Discussion

The results of this retrospective cohort study provide strong evidence of direct causal relationship between non-therapeutic genital cutting and the subsequent development of PTSD-like symptoms among Filipino boys. Destructive, non-therapeutic genital cutting of defenceless minors is a serious abuse of power enacted on children by attacking the root of their sexual identity. In males, circumcision irreversibly diminishes and damages their sexual organ with the guaranteed result of significantly reducing sexual sensation. Consequently, many young circumcised men suffer from premature ejaculation [26,27], while many circumcised middle aged and older adult men suffer from delayed ejaculation and reduced sexual performance, and many also are victims of erectile dysfunction [12,[22], [23], [24], [25], [26], [27]]. In a recent exposé published in the prominent *Psychology Today* magazine, Narvaez warned that,

The circumcision of children has myriad negative psychological consequences …. Removing healthy tissue in the absence of any medical need harms the patient and is a breach of medical providers' ethical duty to the child …. all people have a right to bodily autonomy and self-determination and [we] deeply respect this fundamental tenet of international human rights law (UNESCO 2005) [32,33].

This large-scale retrospective cohort study empirically documents the clearcut psychosexual harm caused by both ritual and medical non-therapeutic genital cutting procedures. In this supposedly enlightened twenty-first century, destructive genital cutting of non-consenting minors, carried out as a rite of passage, is patently unethical and the time has come to recognize once and for all that children's bodily integrity is a fundamental human right that must be respected. The societal pressure exerted on young healthy boys in the Philippines to endure destructive non-therapeutic genital cutting is clearly a flagrant abuse of power and violation of children's human rights [34,35].

### 4.1 Limitations

The lack of a control group of genitally intact boys makes determining a baseline of PTSD in the male population aged between 11 and 16 years uncertain thereby making the observed results somewhat difficult to contextualise. As well, the different sample sizes of the two comparison groups (and the resultant heterogeneity of variance) may well have contributed to higher levels of statistical significance than otherwise might have been observed. Future research should analyse the severity of reported PTSD-like symptoms taking into account changing attitudes and social changes in the Filipino boys’ lives, how this affects their perceptions of genital cutting, and whether as adults they would also subject their own children to these destructive genital-cutting procedures. Replicating the present study among other populations and regions of the Philippines would be instructive in helping to provide external validation. Although both medically-performed and ritual genital cutting is a controversial topic, given widely held subjective views among Filipinos based on unchallenged cultural and religious beliefs, it is time to consider the human rights of Filipino boys and to cease this physically, sexually, and psychologically damaging practice.

## Provenance and peer review

Not commissioned, externally peer reviewed.

## Ethical approval

Ethical approval was granted by Bond University Human Research Ethics Committee.

## Sources of funding

No sources of funding

## Conflicts of interest

No conflicts of interest.

## Author contribution

The brief commentary was written entirely by Gregory J. Boyle.Samuel Ramos conducted the study under the supervision of Gregory J. Boyle.

## Research registration number

Registration number: **ACTRN12619000589189**.

http://www.anzctr.org.au/TrialSearch.http://www.anzctr.org.au/TrialSearch

## Guarantor

Gregory J. Boyle.

## Appendix A Supplementary data

Multimedia component 1 Multimedia component 1

Supplementry Supplementry
